# Cdk5-mediated inhibition of APC/C-Cdh1 switches on the cyclin D1-Cdk4-pRb pathway causing aberrant S-phase entry of postmitotic neurons

**DOI:** 10.1038/srep18180

**Published:** 2015-12-10

**Authors:** Miguel Veas-Pérez de Tudela, Carolina Maestre, María Delgado-Esteban, Juan P. Bolaños, Angeles Almeida

**Affiliations:** 1 Institute of Biomedical Research of Salamanca (IBSAL), University Hospital of Salamanca – University of Salamanca, 37007 Salamanca, Spain; 2 Institute of Functional Biology and Genomics (IBFG), University of Salamanca - CSIC, 37007 Salamanca, Spain

## Abstract

The anaphase-promoting complex/cyclosome (APC/C) is an E3 ubiquitin ligase that regulates cell cycle progression in proliferating cells. To enter the S-phase, APC/C must be inactivated by phosphorylation of its cofactor, Cdh1. In post-mitotic cells such as neurons APC/C-Cdh1 complex is highly active and responsible for the continuous degradation of mitotic cyclins. However, the specific molecular pathway that determines neuronal cell cycle blockade in post-mitotic neurons is unknown. Here, we show that activation of glutamatergic receptors in rat cortical primary neurons endogenously triggers cyclin-dependent kinase-5 (Cdk5)-mediated phosphorylation of Cdh1 leading to its cytoplasmic accumulation and disassembly from the APC3 core protein, causing APC/C inactivation. Conversely, pharmacological or genetic inhibition of Cdk5 promotes Cdh1 ubiquitination and proteasomal degradation. Furthermore, we show that Cdk5-mediated phosphorylation and inactivation of Cdh1 leads to p27 depletion, which switches on the cyclin D1-cyclin-dependent kinase-4 (Cdk4)-retinoblastoma protein (pRb) pathway to allow the S-phase entry of neurons. However, neurons do not proceed through the cell cycle and die by apoptosis. These results indicate that APC/C-Cdh1 actively suppresses an aberrant cell cycle entry and death of neurons, highlighting its critical function in neuroprotection.

Neurons are post-mitotic cells that remain resting in a quiescent G0 phase due to an active down-regulation of cell cycle related proteins. However, increasing evidence indicates that progressive neuronal death associated with neurodegenerative diseases is consequence of an attempt of post-mitotic neurons to aberrantly enter the cell cycle^1^. Thus, in damaged brain areas from preclinical and mild Alzheimer’s disease, it has been observed the expression of cell cycle genes that have been proposed to precede neuronal loss^1^,^2^,^3^,^4^. Furthermore, cell cycle entry has also been described in acute brain injury following ischemic stroke^5^,^6^,^7^,^8^. Although the pathophysiology of both acute and chronic neurological disorders has not yet been elucidated^9^,^10^, the excessive stimulation of glutamatergic receptors (excitotoxicity) is widely accepted. However, whether during excessive glutamatergic stimulation, neurons undergo cell cycle entry, is still unclear.

Recently, we reported that APC/C-Cdh1 activity, which regulates cell-cycle progression in proliferating cells^11^,^12^, is essential for neuronal survival^13^,^14^, thus linking proliferation to neurodegeneration. Furthermore, Cdh1 phosphorylation at Cdk sites promotes cell entry into a new S-phase in proliferating cells^15^,^16^,^17^ and mediates excitotoxic cell death in post-mitotic neurons^14^,^18^. Yet, it is unknown whether phosphorylated Cdh1 triggers aberrant cell cycle entry in post-mitotic neurons. Here we describe that, upon excessive glutamatergic stimulus resembling excitotoxicity, phosphorylated Cdh1 accumulated in the cytoplasm and failed to associate with the APC3 core protein, leading to the inactivation of APC/C in neurons. This occurred through a Cdk5-dependent mechanism that reduced p27 levels, switching on a cyclin D1-Cdk4-pRb pathway that led to S-phase entry and neuronal apoptosis.

## Results and Discussion

### Glutamate-induced Cdh1 phosphorylation disassembles Cdh1 from APC3 leading to APC/C inactivation

**Figure 1:**
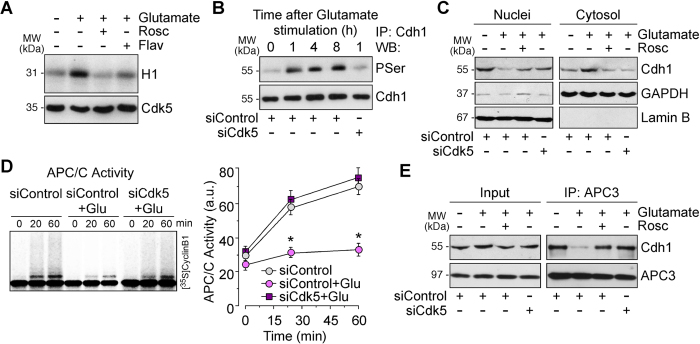
Glutamate-induced Cdh1 phosphorylation disassembles Cdh1 from APC3 leading to APC/C inactivation. Rat cortical neurons were treated with glutamate (100 μM, 5 min) and were further incubated in culture medium, supplemented with Cdk inhibitors, 10 μM roscovitine (Rosc) and 1 μM flavopiridol (Flav), for 1–20 h. When indicated, neurons on day 4 *in vitro* were transfected with a siRNA against luciferase (siControl; 100 nM) or with siRNA against Cdk5 (siCdk5; 100 nM) for 3 days and then treated with glutamate (**A**) At 1 hour after glutamate stimulation Cdk5 was activated in neurons, which was prevented by Rosc. (**B**) Glutamate stimulation triggered Cdh1 phosphorylation in a time-dependent manner. Knockdown of Cdk5 (siCdk5-treated neurons) prevented Cdh1 phosphorylation in glutamate-treated neurons. (**C**) Cytosol and nuclei fractions were extracted from neuronal lysates by centrifugation. Cytosolic and nuclear extracts were analyzed by Western blots using antibodies against Cdh1, followed by Lamin B as nuclear marker and Glyceraldehyde 3-phosphate dehydrogenase (GAPDH) as cytosolic marker. (**D**) Glutamate triggered APC/C inactivation at 4 h after treatment, as assessed by the ability of neuronal extracts to ubiquitylate, *in vitro*, ^35^S-cyclin B1. Transfection with siCdk5 prevented glutamate-induced APC/C inactivation. APC/C activity was expressed as densitometry of the bands. (**E**) At 4 h after glutamate stimulation, neuronal extracts were obtained, immunoprecipitated with anti-APC3 antibody, and analyzed by Western blot for Cdh1 and APC3. Of the whole cellular extracts used for immunoprecipitation, 10% were loaded on SDS-PAGE as an input control. In A, B, C, E a representative western blot is shown out of three. The relative protein abundance is shown in Supplementary Fig. 1. In D data are expressed as means ± S.E.M. (n = 3 independent neuronal cultures). *p < 0.05 *versus* siControl neurons.

To investigate the molecular mechanisms responsible for cell cycle activation in postmitotic neurons following glutamatergic stimulation, neurons were incubated with 100 μM glutamate for 5 min, and then harvested at different time points. We used this procedure as it has been previously demonstrated that it activates an endogenous calcium-dependent signalling cascade^19^ leading to Cdk5 activation^18^. As shown in Fig. 1, glutamate treatment triggered roscovitine- and flavopiridol-inhibitable H1 phosphorylation *in vitro* (Fig. 1A, Supplementary Fig. 1A) and a time-dependent, siCdk5-inhibitable phosphorylation of Cdh1 (Fig. 1B, Supplementary Fig. 1B). This result is not unexpected, since we previously identified at least three Cdk5-dependent phosphorylation sites in Cdh1^18^, namely Ser40, Thr121 and Ser151 that were recently confirmed in the atomic structure of human APC/C-Cdh1^20^. Moreover, here we found that Cdh1 was mainly located in the nucleus (Fig. 1C, Supplementary Fig. 1C); however, glutamate induced Cdh1 release from the nucleus to the cytosol through a Cdk5-mediated mechanism (Fig. 1C, Supplementary Fig. 1C). Since Cdh1 phosphorylation by cyclin-dependent kinases (Cdk) sites is known to cause APC/C inactivation in yeast and in dividing cells^15^,^16^,^21^, we sought to determine APC/C activity in glutamate-treated neurons. As shown in Fig. 1D, glutamate stimulation inhibited APC/C activity, as judged by decreased ubiquitination of its cognate substrate, cyclin B1, an effect that was prevented by siCdk5, indicating a Cdk5-mediated effect. In view that phosphorylation of Cdh1 at Cdk5 sites has been hypothesized to destabilize Cdh1 interaction with the human APC/C complex core protein APC3^20^, we next assessed this possibility under our conditions. To perform this, we immunoprecipitated APC3 in neuronal extracts and APC3 immunoprecipitates were immunoblotted against Cdh1. As revealed in Fig. 1E, APC3-Cdh1 interaction was abolished after glutamatergic stimulation, an effect that was prevented by both inhibiting Cdk with roscovitine and knocking down Cdk5 (Supplementary Fig. 1D). These results indicate that glutamatergic stimulation causes Cdk5-mediated Cdh1 phosphorylation, disrupting APC/C-Cdh1 interaction leading to enzyme inactivation.

### APC/C inhibition causes Cdh1 protein stabilization upon glutamatergic stimulation

**Figure 2:**
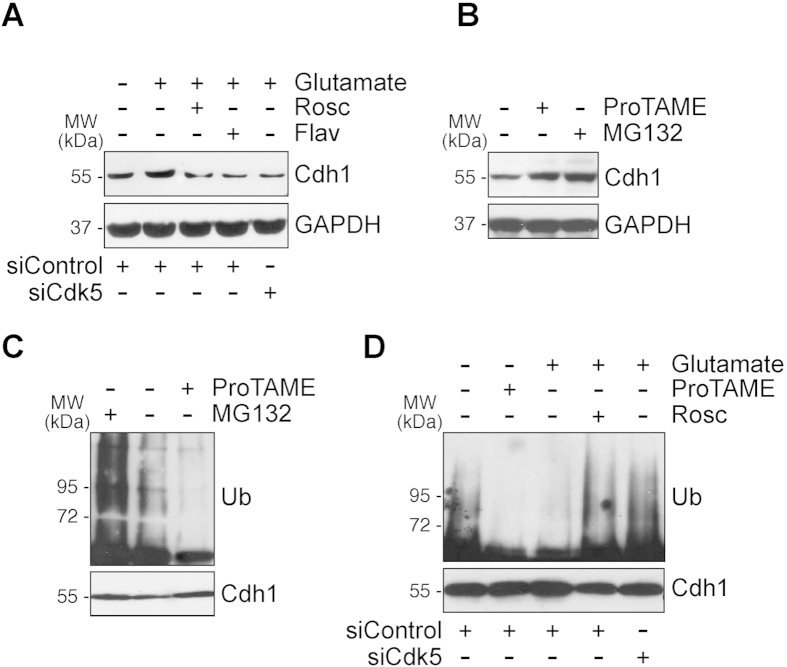
Cdk5 phosphorylates Cdh1 and triggers APC/C inhibition causing Cdh1 protein stabilization. (**A**) Rat cortical neurons on day 4 *in vitro* were transfected with a siRNA against luciferase (siControl; 100 nM) or with siRNA against Cdk5 (siCdk5; 100 nM) for 3 days. Neurons were then treated with glutamate (100 μM, 5 min) and were further incubated in culture medium, supplemented with Cdk inhibitors, 10 μM roscovitine (Rosc) and 1 μM flavopiridol (Flav), APC/C inhibitor, ProTAME (10 μM), and proteasome inhibitor, MG132 (10 μM), for 4 h. Cdh1 protein levels were analyzed by Western blotting. GAPDH protein levels were used as loading control (**B**) Neuronal were treated with ProTAME (10 μM), and MG132 (10 μM), for 4 h, and Cdh1 protein levels were analyzed by Western blotting. (**C**) Neuronal extracts were immunoprecipitated with anti-Cdh1 antibody and analyzed by Western blot for ubiquitin (Ub). (**D**) Rat cortical neurons were treated as in (**A**) and neuronal extracts were immunoprecipitated with anti-Cdh1 antibody and analyzed by Western blot for ubiquitin (Ub). In all cases a representative western blot is shown out of three. The relative protein abundance is shown in Supplementary Fig. 1.

Besides the changes in Cdh1 phosphorylation affecting APC/C activity, it has been proposed that Cdh1 protein stability is amenable to regulation by APC/C activity during the cell cycle^22^. Thus, during mitosis, APC/C is inactive and Cdh1 protein levels are high; however, during the G0/G1 phase, APC/C is active and Cdh1 protein levels are low, albeit still sufficient to bind, and activate, APC/C. However, whether Cdh1 protein levels are subjected to regulation in post-mitotic neurons is unknown. Interestingly, we show that endogenous Cdh1 protein levels increased upon glutamatergic stimulation, an effect that was mediated by Cdk5 (Fig. 2A, Supplementary Fig. 1E). Since Cdk5 phosphorylates Cdh1 (our results), and it has previously been shown that Cdk-mediated Cdh1 phosphorylation stabilizes Cdh1 protein^23^, our data strongly suggest that Cdh1 protein accumulation upon glutamatergic stimulation takes place after Cdk5-mediated Cdh1 phosphorylation. Furthermore, Cdh1 protein was stabilized in post-mitotic neurons by inhibiting APC/C activity with ProTAME or by inhibiting the proteasome with MG132 (Fig. 2B, Supplementary Fig. 1F). In good agreement with this, MG132 triggered an accumulation of ubiquinitated forms of Cdh1, whereas ProTAME decreased Cdh1 ubiquitination (Fig. 2C, Supplementary Fig. 1G), indicating that Cdh1 protein stability is regulated by the APC/C-proteasome pathway. To further test if Cdh1 protein levels are amenable to regulation by an endogenous stimulus, we next assessed Cdh1 stability upon glutamatergic activation. As shown in Fig. 2D, glutamatergic stimulation mimicked ProTAME at inhibiting Cdh1 ubiquitylation, an effect that was prevented by inhibiting Cdk with roscovitine or knocking down Cdk5 (Supplementary Fig. 1H). Altogether, these data indicate that, upon glutamatergic stimulation, Cdk5 phosphorylates Cdh1, which triggers a positive loop of APC/C inhibition leading to the accumulation of inactive (phosphorylated) Cdh1.

### Glutamatergic stimulation causes p27 depletion and activation of the cell cycle machinery in postmitotic neurons through a Cdk5-mediated mechanism

**Figure 3:**
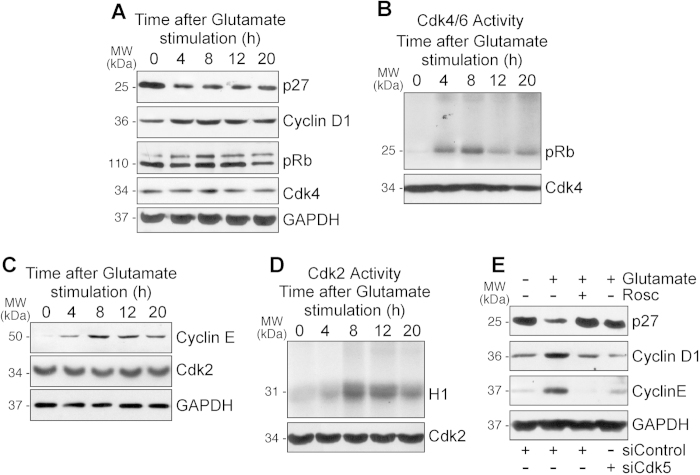
Glutamatergic stimulation causes p27 depletion and activation of the cell cycle machinery in postmitotic neurons through a Cdk5-mediated mechanism. Rat cortical neurons were treated with glutamate (100 μM, 5 min) and were further incubated in culture medium for 4–20 h. Levels of (**A**) p27, cyclin D1, retinoblastoma protein (pRb), and Cdk4 and (**C**) levels of cyclin E and Cdk2 were detected by Western blotting. GAPDH protein levels were used as loading control. (**B**,**D**) Neuronal extracts were immunoprecipitated with anti-Cdk4 (**B**) and anti-Cdk2 (**D**) antibodies. Kinase activities were assayed as the ability to phosphorylate pRb (1 mg/ml), for Cdk4, and histone H1 (1 mg/ml), for Cdk2, *in vitro*. Samples were subjected to SDS-polyacrylamide gel (12%) electrophoresis and transferred proteins were visualized by autoradiography or blotted with anti-Cdk4 and anti-Cdk2. (**E**) Rat cortical neurons were transfected with siRNA against Cdk5 (100 nM siCdk5) for 3 days. Neurons were then treated with glutamate (100 μM, 5 min) and were further incubated in culture medium, supplemented with 10 μM roscovitine (Rosc), for 8 hours. Inhibition of Cdk5 activation by both roscovitine treatment and siCdk5 transfection prevented changes in cells cycle proteins (p27, cyclin D1 and cyclin E) induced by excitotoxicity. In all cases, a representative western blot is shown out of three. The relative protein abundance is shown in Supplementary Fig. 2.

Given our data indicating APC/C inactivation by Cdh1 phosphorylation in post-mitotic neurons, and that it is known that APC/C-Cdh1 inhibition promotes S-phase in dividing cells^15^,^16^,^17^, we next asked whether post-mitotic neurons entered into the cell cycle upon the glutamatergic stimulus. As shown in Fig. 3A, protein levels of the Cdk inhibitor p27 decreased (Supplementary Fig. 2A), whereas those of cyclin D1 increased after glutamatergic stimulus (Supplementary Fig. 2B); cyclin D1 partner, Cdk4, remained unchanged (Fig. 3A, Supplementary Fig. 2B). Since these events are compatible with cyclin D1-Cdk4 activation, we next assessed the levels of its substrate, pRb. Indeed, we found a time-dependent increase in pRb phosphorylation in neurons upon glutamatergic stimulation (Fig. 3A, Supplementary Fig. 2A). Moreover, the ability of neuronal protein extracts to phosphorylate pRb *in vitro* also increased time-dependently (Fig. 3B, Supplementary Fig. 2C). Thus, glutamatergic stimulation promotes cyclin D1-Cdk4-mediated pRb phosphorylation in post-mitotic neurons, a typical feature of cells exiting from the quiescent status and progressing through the G1-phase restriction point of the cell cycle^24^,^25^. To further confirm this issue, we next determined the protein levels of cyclin E as an index of E2F transcriptional activity. Thus, E2F is sequestered inactive by hypophosphorylated pRb; however, upon Cdk-mediated pRb phosphorylation, E2F is released, promoting the transcriptional activation of S-phase genes, including cyclin E, that are required for cell cycle progression^26^. As shown in Fig. 3C, we found that cyclin E increased in neurons upon glutamatergic stimulation, being the levels of cyclin E partner, Cdk2, maintained (Fig. 3C, Supplementary Fig. 2D); these results strongly suggest cyclin E-Cdk2 activation. To confirm this directly, we determined Cdk2 activity in nuclear extracts of neurons after glutamatergic stimulation, and we found a time-dependent increase as judged by their ability to phosphorylate H1 (Fig. 3D, Supplementary Fig. 2E). This suggests that active cyclin E-Cdk2 is responsible for the observed maintenance of pRb in its phosphorylated status after the glutamatergic stimulation (Fig. 3A), known to be necessary to drive the progression of proliferating cells into the S-phase^27^. Finally, we found that the decrease in p27 (Fig. 3E, Supplementary Fig. 2F), and the increases in cyclin D1 (Fig. 3E, Supplementary Fig. 2G) and cyclin E (Fig. 3E, Supplementary Fig. 2H) protein levels, were prevented by Cdk5 knockdown and Cdk inhibition by roscovitine, confirming the upstream role of Cdk5 in this molecular activation of the cell cycle machinery in post-mitotic neurons.

### Phenotypic evidence for aberrant cell cycle entry of post-mitotic neurons triggered by the Cdk5-phospho-Cdh1 pathway upon glutamatergic stimulation

**Figure 4:**
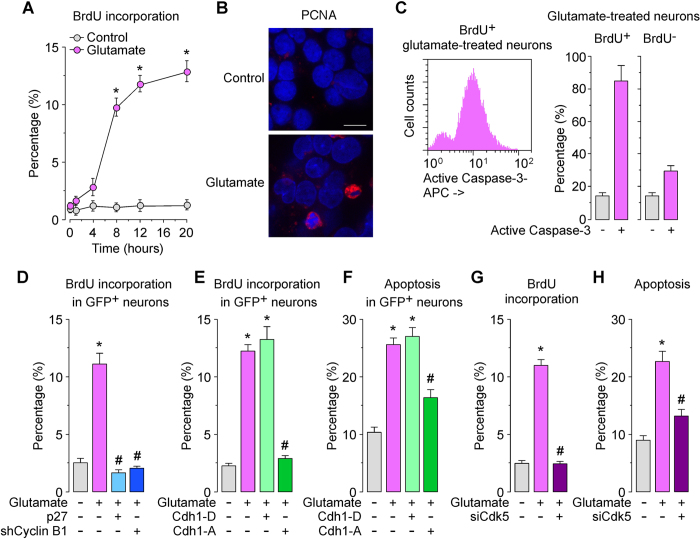
Cdk5-mediated Cdh1 phosphorylation induces cell cycle entry leading to neuronal apoptosis in excitotoxicity. Rat cortical neurons were treated with glutamate (100 μM, 5 min) and were further incubated in culture medium for 4–20 h. (**A**) Bromo-d-Uridine (BrdU) incorporation was measured by flow cytometry. (**B**) At 20 h after glutamate treatment, PCNA (proliferating cell nuclear antigen) was detected by immunocytochemistry. Microphotographs reveal that glutamate induced nuclear PCNA expression in neurons. Scale bar = 10 μM. (**C**) Immediately after glutamate stimulation, neurons were incubated in culture medium containing 10 μg/mL BrdU, for 20 hours. Neurons were then immunostained with BrdU and active caspase-3, which were detected by flow cytometry. Most (85%) of the BrdU^+^ neurons cells expressed active caspase-3. (**D**) Neurons were transfected with pSuper-neo.gfp-Cyclin B1 (expressing both shCyclin B1 and the enhanced GFP) or pIRES2-EGFP mammalian expression vector co-expressing p27 and the enhanced GFP. Control cells were transfected with pSuper-neo.gfp (shControl). Neurons were then treated with glutamate (100 μM, 5 min) and were further incubated in culture medium for 20 h. Flow cytometry analysis for BrdU incorporation was performed in transfected (identified by GFP fluorescence) neurons. (**E**,**F**) Neurons were transfected with pIRES2-EGFP mammalian expression vector co-expressing the enhanced GFP and either the phosphomimetic (Cdh1-P) or phosphodefective (Cdh1-A) forms of Cdh1. Control cells were transfected with empty vector (pIRES2-EGFP). Neurons were then treated with glutamate (100 μM, 5 min) and were further incubated in culture medium for 20 h. BrdU incorporation and neuronal apoptosis were measured in GFP^+^ (transfected) neurons by flow cytometry. (**G**, **H**) Neurons were transfected with siRNA against luciferase (100 nM; siControl) or with siRNA against Cdk5 (100 nM; siCdk5) for 3 days. Neurons were then treated with glutamate (100 μM, 5 min) and were further incubated in culture medium, for 20 hours. BrdU incorporation and neuronal apoptosis were measured by flow cytometry. In all cases, the represented values are means ± S.E.M. (n = 3 independent neuronal cultures). *p < 0.05 *versus* untreated (Control) neurons. ^#^p < 0.05 *versus* Glutamate-treated neurons.

In order to confirm that neurons actually entered the cell cycle, we performed a BrdU incorporation assay. Glutamatergic stimulation time dependently increased BrdU incorporation (Fig. 4A) and induced the nuclear accumulation of the S-phase marker, PCNA (Proliferating cell nuclear antigen), (Fig. 4B) in neurons. Interestingly, we noticed that neurons expressing PCNA showed condensed and fragmented nuclei (Fig. 4B), suggesting apoptotic cell death. To confirm this directly, we next analyzed the proportion of cell cycle-entering neurons –i.e., BrdU^+^ neurons– having signs of apoptosis. Flow cytometry analysis revealed that most BrdU^+^ neurons (85%) co-expressed the apoptotic marker, active caspase-3 (Fig. 4C). Conversely, we found that the expression of active caspase-3 was significantly lower in neurons that failed to enter the cell cycle –i.e., BrdU^-^ neurons– (Fig. 4C). This result is consistent with recent findings^28^ showing neuronal death upon forced S-phase re-entry of hippocampal neurons by Cdk4. However, here we also sought to ascertain the role played by p27 destabilization in such S-phase entry of post-mitotic neurons. Thus, p27 is destabilized upon phosphorylation by cyclin B1-Cdk1, cyclin A-Cdk2, and cyclin E-Cdk2^29^,^30^,^31^. In addition, we have demonstrated that inhibition of APC/C-Cdh1 triggers cyclin B1-Cdk1 activation leading to p27 decrease^32^,^33^, and that Cdk5-mediated Cdh1 phosphorylation causes cyclin B1-Cdk1 activation^34^ in neurons. We therefore aimed to ascertain whether this cyclin B1-Cdk1-p27 pathway could mediate the incorporation of BrdU into neurons upon glutamatergic stimulation. As shown in Fig. 4D, knockdown of cyclin B1 abrogated the S-phase entry, an effect that was mimicked with over-expressing the Cdk inhibitor p27. These results are compatible with the notion that glutamatergic-mediated APC/C-Cdh1 inhibition leading to cyclin B1-Cdk1 activation is responsible for decreased p27 levels leading to S-phase entry of post-mitotic neurons. To test this possibility, we next directly assessed the involvement of Cdh1 phosphorylation status in cell cycle entry. To do this, post-mitotic neurons were transfected with either the phosphomimetic (Cdh1-D) or the phosphodefective (Cdh1-A) form of Cdh1, and then subjected to glutamatergic stimulation. To assess cell cycle entry and apoptosis, we analyzed BrdU incorporation and the percentage of annexin V-positive/7-aminoactinomycin D (7-AAD)-negative (apoptotic) neurons, by flow cytometry, 20 h after the glutamatergic stimulation. Our results show that expression of the phosphomimetic form of Cdh1 (Cdh1-D) had no effect on glutamate-induced BrdU incorporation (Fig. 4E) and neuronal apoptotic death (Fig. 4F), as expected. In contrast, expression of a phosphodefective form of Cdh1 (Cdh1-A) significantly prevented glutamate-induced BrdU incorporation (Fig. 4E) and neuronal apoptotic death (Fig. 4F). To investigate the involvement of Cdk5 in this pathway, we knocked down Cdk5 (siCdk5), which abrogated both BrdU incorporation (Fig. 4G) and apoptotic death (Fig. 4H). These results indicate that Cdk5-mediated phosphorylation of Cdh1 triggers the aberrant cell cycle entry upon glutamatergic stimulation of post-mitotic neurons.

In conclusion, here we show that glutamatergic stimulation down-regulates p27 levels leading to the activation of the cyclin D1-Cdk4-pRb cell cycle pathway, thus triggering an aberrant S-phase entry of post-mitotic neurons. These results therefore reveal the molecular pathway responsible for the well-known observation that neurons of the degenerating areas of neurological diseases show signs of cell cycle entry^1^,^2^,^3^,^4^,^5^,^6^,^7^,^8^. Glutamatergic over-stimulation in neurons is a common feature of neurodegenerative diseases, such as Alzheimer’s disease^9^,^35^ and stroke^10^, hence these results directly identify several potential therapeutic targets useful for the designing of novel strategies against these devastating diseases.

## Methods

### Ethical statement regarding the use of animals

All animals used in this work were obtained from the Animal Experimentation Service of the University of Salamanca, in accordance with Spanish legislation (RD 1201/2005) under license from the Spanish Government. Protocols were approved by the Bioethics Committee of the University of Salamanca.

### Cell cultures

Primary cultures of rat cortical neurons were prepared from fetal Wistar rats of 16 days of gestation^18^, seeded at 2.5 × 10^5^ cells/cm^2^ in different size plastic plates coated with poly-D-lysine (15 µg/ml) and incubated in Dulbecco’s Modified Eagle’s Medium (DMEM) (Sigma, Madrid, Spain) supplemented with 10% fetal calf serum (FCS; Roche Diagnostics, Heidelberg, Germany). Cells were incubated at 37 °C in a humidified 5% CO_2_-containing atmosphere. At 48 hours after plating, the medium was replaced with DMEM supplemented with 5% horse serum (Sigma, Madrid, Spain), 20 mM D-glucose and, on day 4, cytosine arabinoside (10 μM) to prevent non-neuronal proliferation. Cells were used for the experiments on day 6–7 *in vitro*.

### Small interference RNA (siRNA) and plasmid constructions

Specific depletion of Cdk5 was achieved by using small (21 bp) interfering double-stranded ribonucleotides (siRNA) specifically designed to target the coding sequence of the rat Cdk5 mRNA^18^,^34^. We used the following siRNA (only the forward strand shown): 5′-AAGCCGUACCCGAUGUAUC-3′ (nucleotides 859-877 GenBank accession number NM_080885). A siRNA against luciferase (5′-CUGACGCGGAAUACUUCGAUU-3′) was used as control siRNA (siControl). Annealed siRNAs were purchased from Dharmacon (Abgene, Thermo Fisher, Epsom, U.K.). Specific depletion of cyclin B1 was performed by using pSuper-neo.gfp (Oligoengine), including the small hairpin sequences for luciferase (control; 5′-CTGACGCGGAATACTTCGA-3′) or cyclin B1 (shCyclin B1; 5′-GATGGAGCTGATCCAAACC-3′; nucleotides 478-496 GenBank accession number AY338491)^13^,^18^. Human full-length Cdh1 cDNA was a generous gift of Dr J Pines (Gurdon Institute, University of Cambridge, UK), and was used to obtain the Ser-40, Thr-121 and Ser-163 triple Cdh1 mutants using the QuikChange XL site directed mutagenesis kit (Stratagene, La Jolla, CA, USA). Residues were mutated either to Ala (Cdh1-A; to block phosphorylation) or to Asp (Cdh1-D; to mimic a phosphorylated status)^18^.

### Cell transfections and treatments

All transfections with plasmid constructions were performed using Lipofectamine 2000™ (Invitrogen)^18^, following the manufacturer’s instructions. After transfections, cells were further incubated for 1–20 h more until the experiments and cell collection were performed. Transfections of neurons with siRNAs were performed using Lipofectamine RNAiMAX™ (Invitrogen) following the manufacturer’s instructions and used after 72 h.

To promote an excitotoxic insult, neurons were incubated with 100 μM glutamate plus 10 μM glycine in buffered Hank’s solution (134.2 mM NaCl, 5.26 mM KCl, 0.43 mM KH_2_PO_4_, 4.09 mM NaHCO_3_, 0.33 mM Na_2_HPO_4_, 5.44 mM glucose, 20 mM HEPES, 4 mM CaCl_2_, pH 7.4) for 5 min and further incubated in culture medium for the indicated time period^19^. Where indicated, incubations were performed in the presence of 10 μM roscovitine (Rosc; Sigma), 10 μM ProTAME (Boston Biochem, R&D Systems, Minneapolis, USA), or with 10 μM MG132 (Sigma).

### APC/C ubiquitin ligase activity

Active APC/C was immunoprecipitated from neurons using monoclonal anti-APC3 antibody (BD Pharmingen, Becton Dickinson Biosciences) and immobilized on Dynabeads Protein A (Invitrogen, Life Technologies). For ubiquitylation assays, immunoprecipitates were incubated at 37 °C in 10 μl of buffer (0.1 M KCl, 2.5 mM MgCl_2_, 2 mM ATP, 7.5 μg ubiquitin, 0.3 mM dithiothreitol, 135 mM MG132, 1 mM ubiquitin aldehyde, 2.5 mM His-UbcH10 and 2.5 μM UbcH5a in 20 mM Tris-HCl, pH 7.5) containing 2.5 μl of APC/C beads and 1 μl of [^35^S]cyclin B1. Reactions were stopped at the indicated time points with SDS sample buffer, mixtures resolved by SDS-polyacrylamide gel electrophoresis and visualized by phosphorimaging^33^. APC/C activity was measured as densitometry of the bands using ImageJ 1.48u4 software (National Institutes of Health, USA).

### Immunoblots and Immunoprecipitation

Neurons were lysed in 2% sodium dodecylsulphate, 2 mM EDTA, 2 mM EGTA, 50 mM Tris, pH 7.5, supplemented with phosphatase inhibitors (1 mM Na_3_VO_4_, 50 mM NaF) and protease inhibitors (100 μM phenylmethylsulfonyl fluoride, 50 μg/mL anti-papain, 50 μg/mL pepstatin, 50 μg/mL amastatin, 50 μg/mL leupeptin, 50 μg/mL bestatin and 50 μg/mL soybean trypsin inhibitor), stored on ice for 30 min and boiled for 10 min. Aliquots of cell extracts were subjected to SDS polyacrylamide gel (MiniProtean®, Bio-Rad) and blotted with antibodies overnight at 4 °C. Signal detection was performed with an enhanced chemiluminescence kit (Pierce, Thermo Scientific, Waltham, MA, USA). Antibodies used were anti-Cdh1 (AR38, J. Gannon, Clare Hall Laboratories, Cancer Research UK), anti-phosphoserine (ab9332, Abcam), anti-Cdk5 (C-8; sc-173, Santa Cruz Biotechnology, Heidelberg, Germany), anti-GAPDH (Ambion, Cambridge, UK), anti-ubiquitin (ab7780, Abcam, Cambridge, UK), anti-APC3 (35/CDC27; 610455, BD Pharmingen), anti-p27 (57/Kip1/p27; 610242, BD Transduction Laboratories), anti-cyclin D1 (CDS-6; 556470, BD Pharmingen), anti-retinoblastoma protein (pRb; G3-245; 554136, BD Pharmingen), anti-cyclin E (HE12; 551160, BD Pharmingen), anti-Cdk2 (55/Cdk2; 610145, BD Transduction Laboratories), and anti-Cdk4 (97/Cdk4; 610148, BD Transduction Laboratories). For immunoprecipitation, neurons were lysed in ice-cold buffer containing 50 mM Tris (pH 7.5), 150 mM NaCl, 2 mM EDTA, 1% NP-40, supplemented with the phosphatase and protease inhibitors cited above. After clearing debris by centrifugation, neuronal lysates (100 μg) were incubated with anti-Cdh1 for 2 h at 4 °C followed by 1 h incubation with protein G-sepharose (GE Healthcare Life Sciences) at 4 °C. Inmunoprecipitates were extensively washed with lysis buffer and before being resolved by SDS-PAGE and immunoblotted with indicated antibodies^18^.

### Co-immunoprecipitation assay

Neurons were lysed in ice-cold buffer containing 50 mM Tris-HCl, pH 7.5, 150 mM NaCl, 2 mM EDTA, and 1% NP-40, supplemented with the phosphatase and protease inhibitors cited in Western blot analysis. Neuronal extracts were clarified by centrifugation and supernatants (500 μg protein) were incubated with 2 μg anti-APC3 for 4 h at 4 °C, followed by the addition of 30 μl of protein G- sepharose (GE Healthcare) for 2 h at 4 °C. Immunoprecipitates were extensively washed with lysis buffer and detected by Western blot analysis^36^.

### Assays for Cdk activity

Neurons were lysed in ice-cold buffer containing 50 mM Tris (pH 7.5), 150 mM NaCl, 2 mM EDTA, 1% NP-40, supplemented with the phosphatase and protease inhibitors cited above. After clearing debris by centrifugation, extracts (200 μg protein) were incubated with anti-Cdk2 (1 μg) or anti-Cdk4 (1 μg) for 4 h at 4 °C, followed by the addition of 30 μl of protein A-sepharose (GE Healthcare Life Sciences) for 2 h at 4 °C. Immunoprecipitates were washed four times in lysis buffer and resuspended in kinase buffer (50 mM Hepes pH 7.5, 10 mM MgCl_2_, 1 mM EDTA and 0.1 mM dithiothreitol) containing 20 μM ATP, 2 μCi of [γ-32P]ATP and either histone H1 (1 mg/ml; Sigma), for Cdk2, or pRb (1 mg/ml; Calbiochem), for Cdk4. Samples were subjected to SDS-polyacrylamide gel (12%) electrophoresis and transferred proteins were visualized by autoradiography or blotted with either anti-Cdk2 or anti-Cdk4.

### Flow cytometric detection of BrdU

The proportion of neurons entering the S phase was assessed by flow cytometric analysis of bromodeoxyuridine (BrdU) incorporation after 20 h of incubation with 10 μg/ml BrdU, using the APC BrdU Flow kit (Becton Dickinson Biosciences).

### Flow Cytometric detection of apoptotic cell death

Neurons were carefully detached from the plates using 1 mM EDTA (tetrasodium salt) in PBS (pH 7.4) and were stained with annexin V-APC and 7-AAD in binding buffer (100 mM HEPES, 140 mM NaCl, 2.5 mM CaCl_2_) to determine quantitatively the percentage of apoptotic neurons by flow cytometry. Neurons were stained with annexin V-APC and 7-AAD in binding buffer (100 mM HEPES, 140 mM NaCl, 2.5 mM CaCl2) and were analysed on a FACScalibur flow cytometer (15 mW argon ion laser tuned at 488 nm; CellQuest software, Becton Dickinson Biosciences). The annexin V-APC-stained neurons that were 7-AAD-negative were considered to be apoptotic.

### Flow cytometric detection of active caspase-3

After detaching cells with 1 mM EDTA (tetrasodium salt) and centrifuged, cell pellets were fixed during 20 min, re-suspended in PBS + 2% bovine serum albumin (BSA) and incubated for 1 h with anti-active caspase 3 (C92-605; 560901, BD Pharmingen). Cells were then incubated with 1:500 anti-rabbit Cy3 (Jackson Immunoresearch) for 1 h. Between each step, cells were washed with either PBS (until labelling of samples) or PBS + 1% BSA, and re-suspended in PBS + 1% BSA before analysis by flow cytometry (tuned at 488 nm; CellQuest software, BD Biosciences).

### Immunocytochemistry

Neurons grown on glass coverslips were fixed with 4% (vol/vol, in PBS) paraformaldehyde for 30 min and immunostained with anti-PCNA (1:100; 24; 610664, BD Transduction Laboratories). Immunolabeling was deteted by using Alexa 568-conjugated goat anti-mouse (1:500) (Jackson ImmunoResearch) or Alexa 568-conjugated goat anti-rabbit (1:500) (Molecular Probes, Invitrogen). Coverslips were washed, mounted in SlowFade light antifade reagent (Invitrogen) on glass slides, and examined using a microscope (Provis AX70, Olympus) equipped with epifluorescence and appropriated filters sets.

### Protein Determinations

Protein concentrations were determined in the cell suspensions, lysates or in parallel cell culture incubations after solubilization with 0.1 M NaOH. Protein concentrations were determined as described^37^, using bovine serum albumin as standard.

### Statistical analysis

All measurements in cell culture were carried out, at least, in triplicate, and the results are expressed as the mean ± SEM values from at least three different culture preparations. For the comparisons between two groups of values, the statistical analysis of the results was performed by the Student’s *t* test. For multiple values comparisons, we used one-way analysis of variance (ANOVA) followed by Bonferroni test. The statistical analysis was performed using the SPSS 16.0 software for Macintosh. In all cases, *p* < 0.05 was considered significant.

## Additional Information

**How to cite this article**: Veas-Pérez de Tudela, M. *et al.* Cdk5-mediated inhibition of APC/C-Cdh1 switches on the cyclin D1-Cdk4-pRb pathway causing aberrant S-phase entry of postmitotic neurons. *Sci. Rep.* **5**, 18180; doi: 10.1038/srep18180 (2015).

## Supplementary Material

Supplementary Information

## References

[b1] Herrup K. Post-mitotic role of the cell cycle machinery. Curr. Opin. Cell Biol. 25, 711–716 (2013). 24055434 10.1016/j.ceb.2013.08.001 PMC3899578

[b2] Vincent I., Jicha G., Rosado M. & Dickson D. W. Aberrant expression of mitotic cdc2/cyclin B1 kinase in degenerating neurons of Alzheimer’s disease brain. J. Neurosci. 17, 3588–3598 (1997). 9133382 10.1523/JNEUROSCI.17-10-03588.1997 PMC6573674

[b3] Yang Y., Mufson E. J. & Herrup K. Neuronal cell death is preceded by cell cycle events at all stages of Alzheimer’s disease. J. Neurosci. 23, 2557–2563 (2003). 12684440 10.1523/JNEUROSCI.23-07-02557.2003 PMC6742098

[b4] Arendt T., Bruckner M. K., Mosch B. & Losche A. Selective cell death of hyperploid neurons in Alzheimer’s disease. Am. J. Pathol. 177, 15–20 (2010). 20472889 10.2353/ajpath.2010.090955 PMC2893646

[b5] Love S. Neuronal expression of cell cycle-related proteins after brain ischaemia in man. Neurosci. Lett. 353, 29–32 (2003). 14642430 10.1016/j.neulet.2003.09.004

[b6] Erdo F., Trapp T., Mies G. & Hossmann K. A. Immunohistochemical analysis of protein expression after middle cerebral artery occlusion in mice. Acta Neuropathol. 107, 127–136 (2004). 14648078 10.1007/s00401-003-0789-8

[b7] Wen Y. *et al.* Transient cerebral ischemia induces aberrant neuronal cell cycle re-rentry and Alzheimer’s disease-like tauopathy in female rats. J. Biol. Chem. 279, 22684–22692 (2004). 14982935 10.1074/jbc.M311768200

[b8] Rashidian J., Iyirhiaro G. O. & Park D. S. Cell cycle machinery and stroke. B.B.A. 1772, 484–493 (2007). 10.1016/j.bbadis.2006.11.009 17241774

[b9] Dong X. X., Wang Y. & Qin Z. H. Molecular mechanisms of excitotoxicity and their relevance to pathogenesis of neurodegenerative diseases. Acta Pharmacol. Sinica 30, 379–387 (2009). 10.1038/aps.2009.24 PMC4002277 19343058

[b10] Lai T. W., Zhang S. & Wang Y. T. Excitotoxicity and stroke: identifying novel targets for neuroprotection. Prog. Neurobiol. 115, 157–188 (2014). 24361499 10.1016/j.pneurobio.2013.11.006

[b11] Pines J. Cubism and the cell cycle: the many faces of the APC/C. Nat. Rev. Mol. Cell Biol. 12, 427–438 (2011). 21633387 10.1038/nrm3132

[b12] Chang L., Zhang Z., Yang J., McLaughlin S. H. & Barford D. Molecular architecture and mechanism of the anaphase-promoting complex. Nature 513, 388–393 (2014). 25043029 10.1038/nature13543 PMC4456660

[b13] Almeida A., Bolanos J. P. & Moreno S. Cdh1/Hct1-APC is essential for the survival of postmitotic neurons. J, Neurosci. 25, 8115–8121 (2005). 16148219 10.1523/JNEUROSCI.1143-05.2005 PMC6725543

[b14] Almeida A. Regulation of APC/C-Cdh1 and its function in neuronal survival. Mol. Neurobiol. 46, 547–554 (2012). 22836916 10.1007/s12035-012-8309-2 PMC3496556

[b15] Zachariae W., Schwab M., Nasmyth K. & Seufert W. Control of cyclin ubiquitination by CDK-regulated binding of Hct1 to the anaphase promoting complex. Science 282, 1721–1724 (1998). 9831566 10.1126/science.282.5394.1721

[b16] Kramer E. R., Scheuringer N., Podtelejnikov A. V., Mann M. & Peters J. M. Mitotic regulation of the APC activator proteins CDC20 and CDH1. Mol. Biol. Cell 11, 1555–1569 (2000). 10793135 10.1091/mbc.11.5.1555 PMC14867

[b17] Narbonne-Reveau K. *et al.* APC/CFzr/Cdh1 promotes cell cycle progression during the Drosophila endocycle. Development 135, 1451–1461, doi: 10.1242/dev.016295 (2008). 18321983

[b18] Maestre C., Delgado-Esteban M., Gomez-Sanchez J. C., Bolanos J. P. & Almeida A. Cdk5 phosphorylates Cdh1 and modulates cyclin B1 stability in excitotoxicity. EMBO J. 27, 2736–2745 (2008). 18818692 10.1038/emboj.2008.195 PMC2572178

[b19] Almeida A. & Bolaños J. P. A transient inhibition of mitochondrial ATP synthesis by nitric oxide synthase activation triggered apoptosis in primary cortical neurons. J. Neurochem. 77, 676–690 (2001). 11299330 10.1046/j.1471-4159.2001.00276.x

[b20] Chang L., Zhang Z., Yang J., McLaughlin S. H. & Barford D. Atomic structure of the APC/C and its mechanism of protein ubiquitination. Nature 522, 450–454 (2015). 26083744 10.1038/nature14471 PMC4608048

[b21] Jaspersen S. L., Charles J. F. & Morgan D. O. Inhibitory phosphorylation of the APC regulator Hct1 is controlled by the kinase Cdc28 and the phosphatase Cdc14. Curr. Biol. 9, 227–236 (1999). 10074450 10.1016/s0960-9822(99)80111-0

[b22] Listovsky T. *et al.* Mammalian Cdh1/Fzr mediates its own degradation. EMBO J. 23, 1619–1626 (2004). 15029244 10.1038/sj.emboj.7600149 PMC391060

[b23] Huynh M. A., Stegmuller J., Litterman N. & Bonni A. Regulation of Cdh1-APC function in axon growth by Cdh1 phosphorylation. J. Neurosci. 29, 4322–4327 (2009). 19339626 10.1523/JNEUROSCI.5329-08.2009 PMC2839872

[b24] Sherr C. J. G1 phase progression: cycling on cue. Cell 79, 551–555 (1994). 7954821 10.1016/0092-8674(94)90540-1

[b25] Absalon S., Kochanek D. M., Raghavan V. & Krichevsky A. M. MiR-26b, upregulated in Alzheimer’s disease, activates cell cycle entry, tau-phosphorylation, and apoptosis in postmitotic neurons. J. Neurosci. 33, 14645–14659 (2013). 24027266 10.1523/JNEUROSCI.1327-13.2013 PMC3810537

[b26] Dick F. A. & Rubin S. M. Molecular mechanisms underlying RB protein function. Nat. Rev. Mol. Cell Biol. 14, 297–306 (2013). 23594950 10.1038/nrm3567 PMC4754300

[b27] Yoshida A., Yoneda-Kato N. & Kato J. Y. CSN5 specifically interacts with CDK2 and controls senescence in a cytoplasmic cyclin E-mediated manner. Sci. Rep. 3, 1054 (2013). 23316279 10.1038/srep01054 PMC3542532

[b28] Marathe S., Liu S., Brai E., Kaczarowski M. & Alberi L. Notch signaling in response to excitotoxicity induces neurodegeneration via erroneous cell cycle reentry. Cell Death Diff. 22, 1775–1784 (2015). 10.1038/cdd.2015.23 PMC4648324 25822340

[b29] Besson A., Dowdy S. F. & Roberts J. M. CDK inhibitors: cell cycle regulators and beyond. Dev. Cell 14, 159–169 (2008). 18267085 10.1016/j.devcel.2008.01.013

[b30] Yalcin A. *et al.* Nuclear Targeting of 6-Phosphofructo-2-kinase (PFKFB3) Increases Proliferation via Cyclin-dependent Kinases. J. Biol. Chem. 284, 24223–24232 (2009). 19473963 10.1074/jbc.M109.016816 PMC2782016

[b31] Hedblom A. *et al.* CDK1 interacts with RARgamma and plays an important role in treatment response of acute myeloid leukemia. Cell Cycle 12, 1251–1266 (2013). 23518499 10.4161/cc.24313 PMC3674090

[b32] Cuende J., Moreno S., Bolanos J. P. & Almeida A. Retinoic acid downregulates Rae1 leading to APC(Cdh1) activation and neuroblastoma SH-SY5Y differentiation. Oncogene 27, 3339–3344 (2008). 18212744 10.1038/sj.onc.1210987

[b33] Delgado-Esteban M., Garcia-Higuera I., Maestre C., Moreno S. & Almeida A. APC/C-Cdh1 coordinates neurogenesis and cortical size during development. Nat. Commun. 4, 2879 (2013). 24301314 10.1038/ncomms3879

[b34] Veas-Perez de Tudela M. *et al.* Regulation of Bcl-xL-ATP Synthase Interaction by Mitochondrial Cyclin B1-Cyclin-Dependent Kinase-1 Determines Neuronal Survival. J. Neurosci. 35, 9287–9301 (2015). 26109654 10.1523/JNEUROSCI.4712-14.2015 PMC6605197

[b35] Wang Y. & Qin Z. H. Molecular and cellular mechanisms of excitotoxic neuronal death. Apoptosis 15, 1382–1402 (2010). 20213199 10.1007/s10495-010-0481-0

[b36] Gomez-Sanchez J. C. *et al.* The human Tp53 Arg72Pro polymorphism explains different functional prognosis in stroke. J. Exp. Med. 208, 429–437 (2011). 21357744 10.1084/jem.20101523 PMC3058581

[b37] Lowry O. H., Rosebrough N. J., Lewis-Farr A. & Randall R. J. Protein measurement with the Folin phenol reagent. J. Biol. Chem. 193, 265–275 (1951). 14907713

