# Supplementary material for: Cdk5-mediated inhibition of APC/C-Cdh1 switches on the cyclin D1-Cdk4-pRb pathway causing aberrant S-phase entry of postmitotic neurons
Source: Sci Rep. 2015 Dec 10;5:18180. doi: 10.1038/srep18180 (PMC4674757; doi:10.1038/srep18180)
Supplement: Supplementary Information [file srep18180-s1.pdf]

Supplementary Figures

Title: Cdk5-mediated inhibition of APC/C-Cdh1 switches on the cyclin D1-Cdk4-pRb pathway causing aberrant S-phase entry of postmitotic neurons

Authors: Miguel Veas-Pérez de Tudela[1,2], Carolina Maestre[1], María Delgado-Esteban[1,2], Juan P. Bolaños[1,2] and Angeles Almeida[1,2*]

[1]Institute of Biomedical Research of Salamanca (IBSAL), University Hospital of Salamanca – University of Salamanca, 37007 Salamanca, Spain
[2]Institute of Functional Biology and Genomics (IBFG), University of Salamanca-CSIC, 37007 Salamanca, Spain

Corresponding author:
*Angeles Almeida,
Institute of Biomedical Research of Salamanca (IBSAL),
University Hospital of Salamanca – University of Salamanca
Calle Zacarías González, 2
37007 Salamanca
Spain
Phone: +34 923 294 908
Fax: +34 923 224 876
E-mail: aaparra@usal.es

Supplementary Figure 1 - Veas-Perez de Tudela et al

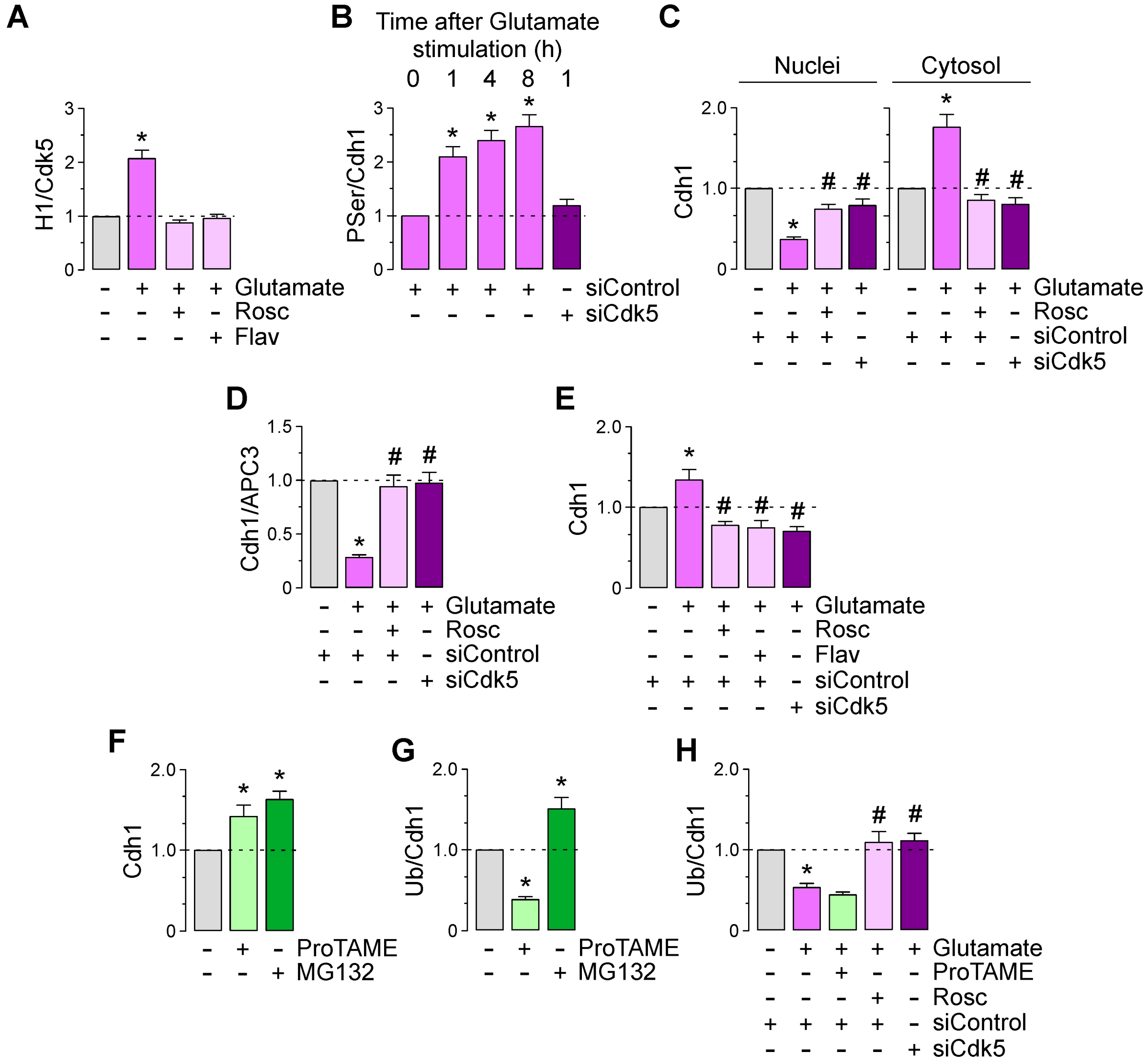

Supplementary Figure 1. Relative protein abundances quantification from results shown in Figs. 1 and 2. (**A**) Results of the relative quantification of protein abundance of Fig. 1A. (**B**) Results of the relative quantification of protein abundance of Fig. 1B. (**C**) Results of the relative quantification of protein abundance of Fig. 1C. (**D**) Results of the relative quantification of protein abundance of Fig. 1E. (**E**) Results of the relative quantification of protein abundance of Fig. 2A. (**F**) Results of the relative quantification of protein abundance of Fig. 2B. (**G**) Results of the relative quantification of protein abundance of Fig. 2C. (H) Results of the relative quantification of protein abundance of Fig. 2D. Data are expressed as means ± S.E.M. (n= 3 independent experiments). *p<0.05 *versus* untreated neurons. #p<0.05 *versus* Glutamate-treated neurons.

Supplementary Figure 2 - Veas-Perez de Tudela et al

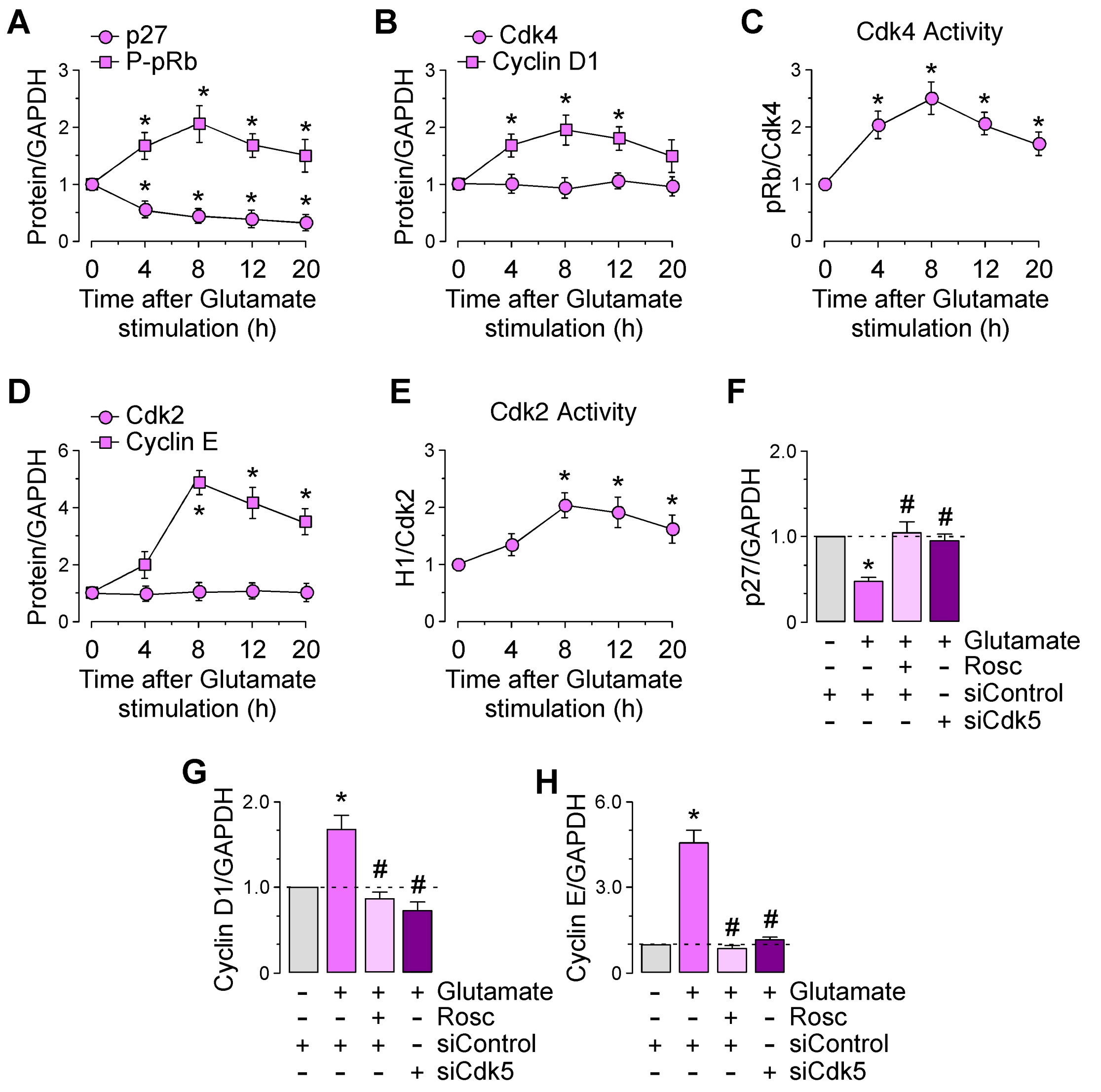

Supplementary Figure 2. Relative protein abundances quantification from results shown in Fig. 3. (**A, B**) Results of the relative quantification of protein abundance of Fig. 3A. (**C**) Results of the relative quantification of protein abundance of Fig. 3B. (**D**) Results of the relative quantification of protein abundance of Fig. 3C. (**E**) Results of the relative quantification of protein abundance of Fig. 3D. (**F, G, H**) Results of the relative quantification of protein abundance of Fig. 3E. Data are expressed as means ± S.E.M. (n= 3 independent experiments). *p<0.05 *versus* 0 hours (A-E) or untreated (F-H) neurons. #p<0.05 *versus* Glutamate-treated neurons.
